# Supplementary figures and images for: Homophily and social mixing in a small community: Implications for infectious disease transmission
Source: PLoS One. 2024 May 28;19(5):e0303677. doi: 10.1371/journal.pone.0303677 (PMC11132460; doi:10.1371/journal.pone.0303677)

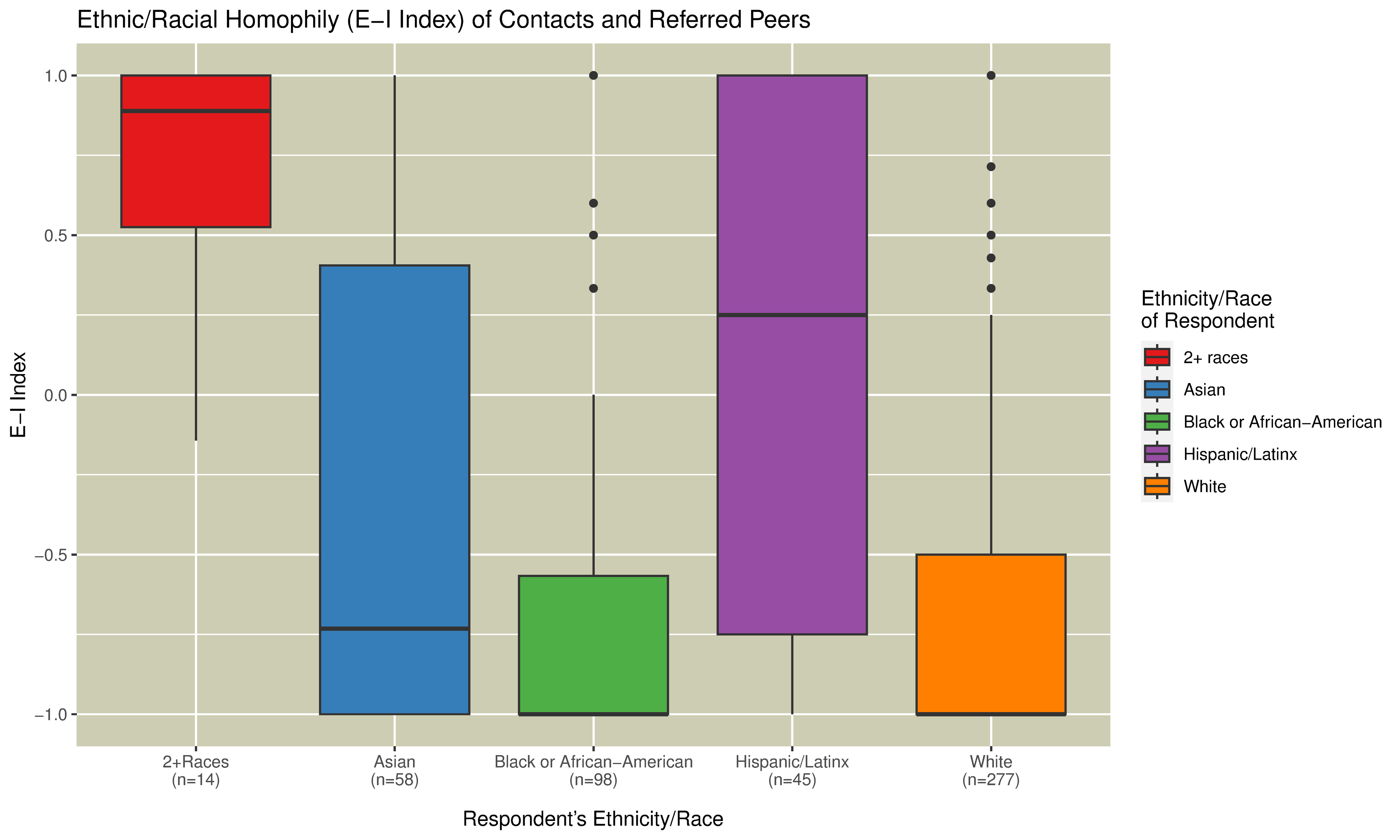

Supplement: S1 Fig — Homophily scores by ethnicity/race, as measured by external-internal (E-I) index, among enrolled seeds (n = 376) and peers (n = 116) who described or recruited at least one person (of the cohort N = 509, 8 seeds and 9 peers did not describe any contacts or recruit anyone else into the study). The unit of analysis for the E-I index is the egocentric network. A score of +1 indicates all out-group ties and a score of -1 indicates all in-group ties. A Kruskal-Wallis test identified significant differences between the groups (χ2(4) = 80.1, p<0.01). A pairwise Dunn test between groups with a Bonferroni correction identified statistically significant differences between all pairs except Black or African-American respondents and White respondents and between Hispanic or Latinx respondents and those who identified as 2 or more ethnicities/races. (TIF) [file pone.0303677.s001.tif]
