# Supplementary material for: Homophily and social mixing in a small community: Implications for infectious disease transmission
Source: PLoS One. 2024 May 28;19(5):e0303677. doi: 10.1371/journal.pone.0303677 (PMC11132460; doi:10.1371/journal.pone.0303677)
Supplement: Attachment — Submitted filename: Review- PLOS ONE-1.docx [file pone.0303677.s002.docx]

PONE-D-23-41812
Social Mixing Patterns Lead to Insights about Infectious Transmission Pathways
PLOS ONE

**Overall impression**

The manuscript engages in an interesting conversation, the nexus between social mixing patterns and infection transmission and promises to project some insightful policy and theoretical implications. However, I think some further clarifications of the following issues may help enhance its potential contribution to policy and theory.

**Introduction**

I believe that the introduction of a research paper needs to present a conversation-problem-solution (contribution) picture of the study.

1) Conversation: what is the scholarly literature that the paper is joining?

2) Problem: what issue in that literature is the paper trying to solve?

3) Solution (contribution): how exactly is the paper addressing that problem?

I see that the authors have projected the conversation they are joining, i.e., the implications of social mixing patterns on infectious (SARS-CoV-2) transmission pathways. They have also indicated in *lines 92-94* that they are joing the conversation *‘To better understand social mixing patterns and infection transmission potential in a small city (Durham County, North Carolina, USA)’*. Certainly, understanding social mixing patterns and infection transmission is important. However, I do not see an established basis for this aim. Is it a knowledge gap to warrant the study? Can the authors point to what is missing in the current literature on the nexus between social mixing patterns and infection transmission?

**Methods**

I am not familiar with the authors’ analytic lens, i.e., egocentric network analysis. However, I think some further explanation of methodological choices can help readers. For instance, why was the study limited to people aged 18 years and older? Why limit the study to only people newly diagnosed with SARS-CoV-2? What does newly diagnosed mean?

I was also wondering how the categorical variables (ethnicity/race and gender) in their online survey were defined? Were they researcher or participant defined? If these were researcher-defined, what standard categorization was used? And why? I believe this is fundamental as there are different and evolving categorizations of these variables.

**Results**

I have no expertise in the kind of analysis presented to enable me provide a useful review comment.

**Discussion**

The authors have provided a detailed discussion of their findings in relation to their research objectives. They have also situated their findings within existing literature. I also see some attempt to point out the strengths and limiations of the study. However, I think a some clarity is needed to point out the concrete policy and theoretical implications of the results. What do policy-makers and health organisations do to manage infection transmission pathways more effectively? And what are new research questions (further research directions) emerge from the findings?
